# Left Ventricular torsion rates by CMR correlate with invasively-derived hemodynamic data in pediatric pulmonary hypertension

**DOI:** 10.1186/1532-429X-18-S1-P4

**Published:** 2016-01-27

**Authors:** Melanie J Dufva, Uyen Truong, Robin Shandas, Vitaly Kheyfets

**Affiliations:** 1 grid.241116.10000000107903411Bioengineering, University of Colorado Denver, Denver, CO USA; 2 grid.413957.d0000000106907621Cardiology, Children's Hospital Colorado, Aurora, CO USA

## Background

Pulmonary hypertension (PH) is a progressive disease that results in right ventricular dysfunction through increased resistive afterload and pulmonary arterial (PA) stiffening. The ventricles are connected at the inter-ventricular sulcus, share an interlaced network of muscle fibers, and are hemodynamically dependent upon each other via the interventricular septum (IVS). Thus, the left ventricle (LV) plays a major role in RV contractile performance and may also be affected in this disease via ventricular-ventricular interaction. The relationship between hemodynamic parameters and torsion rate is unknown. We hypothesized that LV torsion rate is reduced in pediatric PH and is concomitant with hemodynamic markers of PH, and that this is due to the impact of prolonged increased impedance, resulting in reduced RV performance.

## Methods

Tagged cine CMR images were acquired for 8 pediatric PH patients and 18 control-matched subjects. LV systolic torsion rate (TR) was quantified using harmonic phase analysis. Right heart catheterization and echocardiography was performed on PH patients. Vascular ventricular coupling (VVC) was estimated with the single beat pressure-volume method and calculated as the ratio of arterial elastance to ventricular end systolic elastance (contractility).

## Results

LV TR was significantly reduced in PH patients compared to control patients (TR=1.61 ± 0.890/systolic cycle versus 3.04 ± 1.480/systolic cycle, p = 0.0189). LV TR correlated highly with mean pulmonary arterial pressure (mPAP, r = 0.93, p = 0.0008), RV systolic pressure (r = 0.94, p = 0.0005), and pulmonary vascular resistance (PVR, r = 0.93, p = 0.0010). VVC correlated highly with LV TR (r = 0.85, p = 0.0071), as did RV contractility (r = 0.86, p = 0.0063).

## Conclusions

Pediatric PH patients have reduced LV torsion rates compared to those of healthy patients, which could be significantly reducing RV contractile performance. Also, LV TR is strongly related to RV contractility and hemodynamic parameters derived by catheterization.

**Figure 1:**
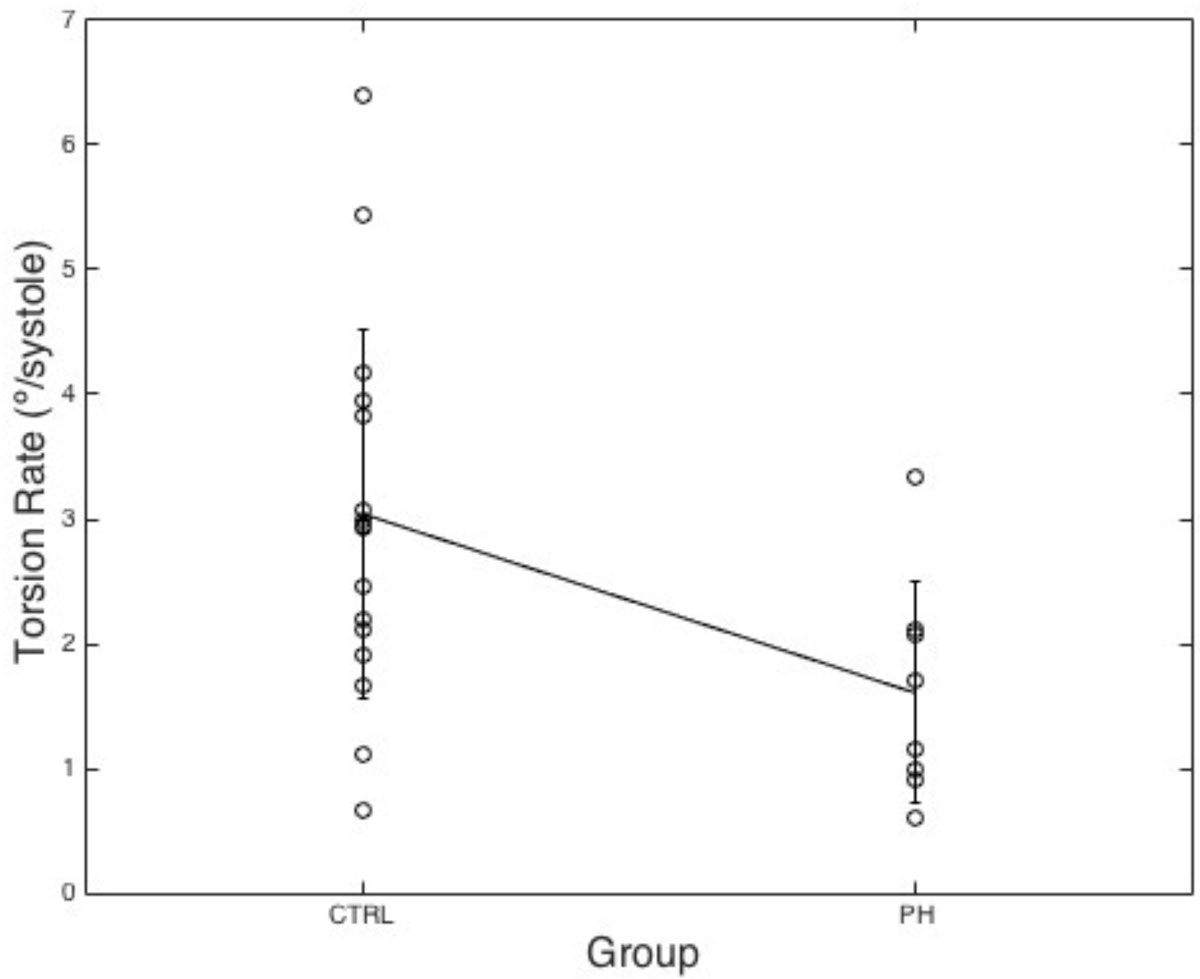
**Torsion rates for PH (1.61 ± 0.89 degrees/systolic cycle) are significantly reduced from control (3.04 ± 1.48 degrees/systolic cycle) patients (p = 0.0189)**.

**Figure 2:**
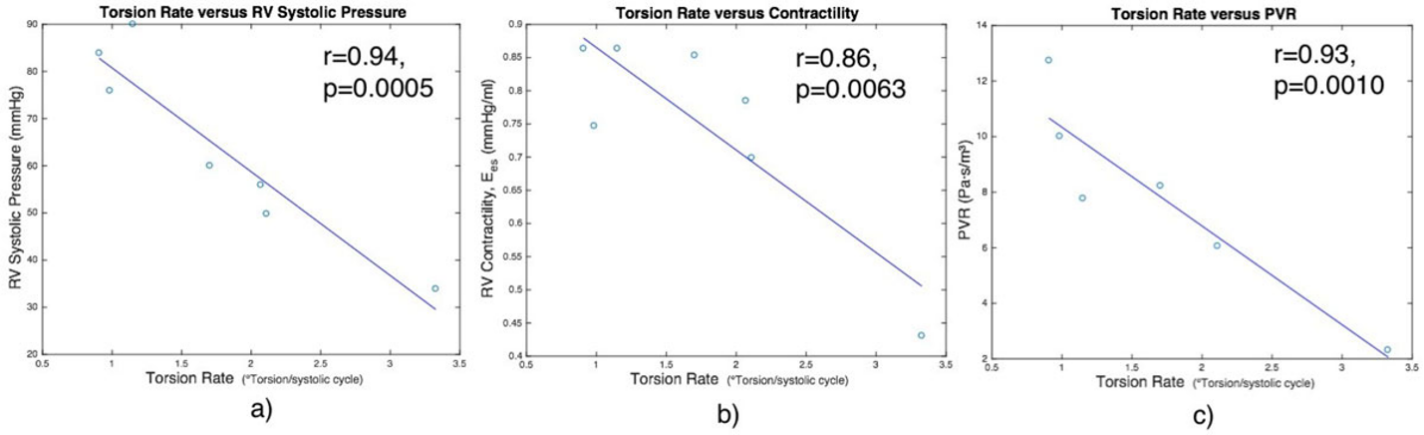
**Torsion rates for PH patients are highly correlated with a) RV systolic pressure (r = 0.94, p = 0.0005), b) RV contractility (r = 0.86, p = 0.0063), and c) PVR (r = 0.93, p = 0.0010)**.

